# Expression of immunophenotypic markers in blood mononuclear cells of patients with advanced head and neck squamous cells carcinoma

**DOI:** 10.1590/acb405425

**Published:** 2025-08-08

**Authors:** Adriana Torres da Silva, Érica Leandro Marciano Vieira, Ana Cristina Simões e Silva, Andy Petroianu

**Affiliations:** 1 Universidade Federal de Minas Gerais - Faculty of Medicine - Department of Surgery - Belo Horizonte (MG), Brazil.

**Keywords:** Carcinoma, Squamous Cell, Head and Neck Neoplasms, Immunophenotyping, Lymphocytes, Leukocytes, Mononuclear

## Abstract

**Purpose:**

To evaluate peripheral blood mononuclear cells (PBMCs) from patients with advanced head and neck squamous cell carcinoma (HNSCC) in comparison with healthy volunteers, as they can be potential biomarkers.

**Methods:**

Immunophenotyping was performed using flow cytometry of blood mononuclear cells from two groups of adult men: group 1 (n = 14), diagnosed with HNSCC (mouth, larynx, and hypopharynx); and group 2 (n = 14), volunteers, healthy, and without the use of drugs. The cell groups studied were T lymphocytes (CD3, CD4, CD8, CD56 and CD69), B lymphocytes (CD19, CD69), neutrophils (CD11a, CD16, CD66b, HLA-DR), and monocytes (CD14, CD86).

**Results:**

In group 1, there were an increase in CD3+CD4+ T lymphocytes (*p* < 0.001) and NK 56+ cells (*p* = 0.009) and a decrease in CD3+CD8+ T lymphocytes (*p* = 0.02) in comparison with group 2. In patients with HNSCC, an increase was found in the expression of the CD69 marker in CD3+CD4+ T lymphocytes (*p* = 0.03) and CD19+ B lymphocytes (*p* = 0.01) when compared to healthy volunteers.

**Conclusion:**

HNSCC triggers a systemic inflammatory response with a decrease in CD8 T cells and an increase in CD4 T and CD56 natural killer cells. CD69 early activation marker was expressed by T and B cells.

## Introduction

The functions of immune system, including actions of lymphocytes, neutrophils, and monocytes, are altered in patients with head and neck squamous cell carcinoma (HNSCC), suggesting that these tumors are immunosuppressive^1^. Studies of the function of T lymphocytes in patients with HNSCC have gained importance due to the advent of immunotherapy^2^,^3^.

The HNSCC cells inhibit the immune system by means of innate and adaptive pro-tumor cells^4^. The HNSCC microenvironment contains suppressor myeloid stromal cells, immature dendritic cells, and T lymphocytes associated with the tumor, whose effect can be favorable to the tumor progression and metastasis. The HNSCC microenvironment contains stromal cells, in addition to neoplastic cells, which include macrophages associated with the tumor, suppressor cells derived from myeloids, immature dendritic cells, and T cells. These immunological cells play an important role in the outcome of the disease. Depending on these cells activation and the surrounding signaling, the effect can be favorable or not to the tumor progression and metastasis^5^.

Based on the outcome from trials with pembrolizumab and nivolumab, Food and Drug Administration approved these immunotherapeutic drugs for the treatment of the metastatic recurrent forms of the disease. Its efficacy is limited to a subgroup of patients and to the definition of biomarkers. Tumors with PD-L1 expression have a greater probability of responding to the inhibitors of the PD-1/PD-L1/PD-L2 axis, although the lack of PD-L1 expression is not an indicator of resistance to PD-1 inhibitors^5^,^6^.

The inflammatory phenotype of T cells is proposed as a response biomarker for immunotherapy^7^. Studies conducted on melanoma suggest that the therapeutic benefits in patients with a pre-existing T-cell response against the tumor, as shown by a CD8+ T cell basal infiltration in the tumor microenvironment^6^,^7^.

There is evidence that signal transduction via CD69 activates the gene expression of proliferating cytokines of CD4+ and CD8+ peripheral T cells, as well as of thymocytes, in addition to inducing the expression of CD25 and interleukin 2 (IL2) dependent cell proliferation^8^,^9^. The CD69’s ability to serve as a signal transduction molecule in various cell systems, coupled with its enhanced expression in certain inflammatory diseases, suggests that it may possibly play a pathogenic role. CD69 has been identified on the surface of lymphocytes, but subsequent studies have documented its expression in various types of cells, suggesting a broad, and perhaps critical, role in cell physiology^8^,^9^. In fact, CD69 has been described as a receptor capable of eliciting multiple cell activation responses. However, the nature of the physiological ligands for CD69 is unclear. Some authors have recently postulated its involvement in the pathogenesis of inflammatory diseases. Such experimental approaches may eventually provide important information about these ligands, as well as on the CD69’s physiological and pathophysiological role^8^,^9^.

Because immunotherapy is indicated in advanced cancer, to understand the immune response at this stage of the disease is necessary. The objective of this study was to evaluate peripheral blood mononuclear cells (PBMCs) from patients with advanced HNSCC in comparison with healthy volunteers, as they can be potential biomarkers.

## Methods

This study was approved by the Research Ethics Committee of Hospital Foundation of the state of Minas Gerais, Brazil, registered under protocol number 075/2009. All participants received explanations about the investigation and were included after agreeing to sign the informed consent form.

This was a study in which PBMC (lymphocytes, monocytes, and neutrophils) were evaluated from two groups of men, 50 to 65 years old:

- Group 1 (n = 14): patients with a mean age of 55 ± 5 years old, all smokers and heavy drinkers, diagnosed with HNSCC of mouth, larynx, and hypopharynx confirmed by incisional biopsy and TNM III and IV staging10, with no other disease and without having be submitted to surgical or adjuvant cancer treatment;
- Group 2 (n = 14): healthy volunteers, with no use of medication and average age of 55 ± 5 years old (Table 1).

**Table 1:** Patients with head and neck squamous cell carcinoma distributed by age, location, and tumor stage.

Patient	Age (years)	Location	Staging
1	65	Mouth	T4N3M0
2	51	Larynx	T4N0M0
3	53	Larynx	T3N2M0
4	51	Mouth	T3N2M0
5	51	Mouth	T3N0M0
6	50	Mouth	T3N2M0
7	62	Larynx	T3N0M0
8	55	Larynx	T4N3M0
9	53	Mouth	T4N3M0
10	53	Mouth	T3N0M0
11	61	Pharynx	T3N0M0
12	60	Mouth	T3N0M0
13	54	Mouth	T3N0M0
14	52	Mouth	T3N0M0

Regarding group 1, HNSCC triggering factors were investigated, including profession, smoking, drinking habits, occupational carcinogens, and reports of allergy (asthma, atopic dermatitis, urticaria, allergic rhinitis and intolerance to dust, animal hair, mold and food). HNSCCs were characterized by their size, location, invaded organs, cervical nodules, and distant metastases through physical examination, direct and indirect laryngoscopy, endoscopy and cervical, chest and abdomen computed tomography. Group 2 volunteers underwent only anamnesis and physical examination of the head and neck. Only men with no apparent disease, who did not use medication, and were not smokers or heavy drinkers were included.

The samples were processed for flow cytometry, immediately after collection, using a FACS Canto II cytometer (Becton & Dickinson, United States of America) capable of reading several parameters simultaneously for each cell, based on their physical peculiarities, granularity, and binding monoclonal antibodies labeled with fluorochrome by means of LASER beam scattering. The cells’ phenotypic, biochemical, and molecular properties characterized the leukocyte, neutrophil, and monocyte populations and subpopulations.

Peripheral blood samples were incubated in the dark at room temperature for 30 minutes with monoclonal antibodies for the following surface markers: anti-CD3, anti-CD4, anti-CD8, anti-CD14, anti-CD86, anti-CD-16, anti-CD66b, anti-CD-11a, anti-HLADR, anti-CD19, anti-CD56, and anti-CD69 (Caltag- Medsystems Limited, United Kingdom), conjugated with phiseriocyanate fluorescein (PE), isothiocyanate (FITC), or biotin. After incubation, erythrocytes were lysed with optilyse-solution B (Immunotec, United States of America). The cells were then washed twice in 1 mL of cold phosphate buffered saline (PBS, pH 7.4). Biotinylated antibodies were revealed using streptavidin, FITC (Begton & Dickinson, United States of America).

The following antibodies were used against human proteins FITC anti-CD3 proteins (clone UCHT1) and FITC anti-CD8 (clone HIT8a) (Bio-Legend, United States of America); anti-CD4 FITC (clone RPA-T4) and anti-CD19 FITC (clone HIB19), and anti-CD56 PE (clone B159), anti-CD19 PE (clone HIB19), anti-CD4 PE (clone L120), and anti-CD8 PE (clone HIT8a) (Begton & Dickinson, United States of America). Cell analysis was processed and analyzed, using Cell Quest software (Begton & Dickinson, United States of America), total T lymphocytes (CD3+), helper T lymphocytes (CD3+ and CD4+), cytotoxic T lymphocytes (CD3+ and CD8+), B lymphocytes (CD3+ and CD19+), cells with NK phenotype (CD3- and CD56+), monocytes (CD14+ and CD86+), and neutrophils (CD11a+, HLA-DR+, CD66b+ and CD16+). The phenotype was analyzed using fluorescence dot plots after selecting the cells of interest population, based on cell size and granularity-graph SSC vs. FSC - FSC: frontal dispersion angle detects cell size. SSC lateral dispersion angle detects cell composition. Each particle that passes through the light beam gives off a different light, thereby differentiating the cell types. The cells were counted using FSC and SSC detectors. They were then analyzed for their expression (frequency and mean fluorescence intensity-MFI) of a given marker, using histograms with markers defined in negative isotype controls.

The descriptive analysis and sample characterization were carried out based on the frequency distribution (absolute and relative) of the selected variables, calculation of mean, standard deviation, medians, and interquartile ranges. For the descriptive analysis, the population was distributed into patients with a confirmed diagnosis of cancer and volunteers without a confirmed diagnosis of cancer. Biomarker results were divided into four sets of categories: lymphocytes, granulocytes, monocytes, and activation markers. Each set was subjected to specific analyses to explore differences between groups and identify relevant patterns. To determine whether the distribution of biomarkers followed a normal pattern, the Shapiro-Wilk’s test was initially applied. This test is widely used to assess the normality of data by comparing the observed distribution with a theoretical normal distribution. When the results indicated a normal distribution, the Student’s t test was applied, an analysis to compare the means of two independent samples, assuming equality of variances. On the other hand, for data that did not present a normal distribution, the Mann-Whitney U test was used. This non-parametric test is ideal for comparing medians of two independent samples, being particularly useful when the data deviates from normality or when the samples are small. Both tests allowed us to evaluate statistical differences in biomarker means or medians between groups of cancer patients and volunteers^11^,^12^.

## Results

The comparison of the percentages of lymphocyte subtypes between patients with HNSCC and healthy volunteers is shown in Table 2. The group of patients with squamous cell carcinoma showed reduction (*p* = 0.03) in the percentage of total lymphocytes compared to healthy volunteers. The results for the percentage of CD56+ cells revealed a difference between the groups (*p* = 0.009), with the group of cancer patients exhibiting a higher mean (14.9%) compared to the group of volunteers (7.8%). In the group of patients with squamous cell carcinoma, the percentage of CD3+CD4+ T lymphocytes was higher (*p* < 0.001), while the percentage of CD3+CD8+ T lymphocytes was lower (*p* = 0.02), when compared to volunteers. Regarding the activation marker CD69, both in CD19+ B lymphocytes and in CD3+CD4+ T lymphocytes, the group with squamous cell carcinoma presented higher percentages, with p indicating differences for CD19+ B lymphocytes (*p* = 0.01) and T lymphocytes CD3+CD4+ (*p* = 0.03).

**Table 2:** Comparison of percentages of subtypes and expression of the CD69 activation marker between patients with head and neck squamous cell carcinoma and healthy volunteers.

Biomarkers	Group	Mean ± SDM	*p* -value
Total lymphocytes	Group 1	20.7 ± 10.0	0.03*
	Group 2	27.4 ± 5.3	
CD3+ lymphocytes	Group 1	67.9 ± 8.1	0.18*
	Group 2	62.4 ± 12.8	
CD56+ lymphocytes	Group 1	14.9 ± 7.1	0.009*
	Group 2	7.8 ± 6.2	
CD69 in B CD19+ lymphocytes	Group 1	38.2 ± 25.2	0.01*
	Group 2	19.1 ± 12.4	
CD69 in CD3+CD8+ lymphocytes	Group 1	32 ± 25.2	0.07**
	Group 2	16.1 ± 9.3	
CD69 in CD3+CD4+ lymphocytes	Group 1	34.2 ± 24.6	0.03*
	Group 2	18.6 ± 10.6	
CD19+ B lymphocytes	Group 1	15.8 ± 12.5	0.2**
	Group 2	11.6 ± 3.0	
CD3+CD8+ lymphocytes	Group 1	20.0 ± 13.5	0.02**
	Group 2	33.2 ± 15.9	
CD3+CD4+ lymphocytes	Group 1	77.2 ± 15.2	< 0.001*
	Group 2	60.4 ± 11.2	

SDM: standard deviation from the mean;

* Student’s t test;

** Mann-Whitney U test.

Table 3 shows the percentages of granulocyte subtypes in the groups of patients and volunteers. Despite the differences observed in the means between the groups, there was no significance. Table 4 shows the percentages of monocyte subtypes in the groups of patients and volunteers. Despite the differences observed in the means between the groups, no difference between the values showed statistical significance.

**Table 3:** Comparison of percentages of granulocyte subtypes between patients with head and neck squamous cell carcinoma and healthy volunteers.

Biomarkers	Group	Mean ± SDM	*p* -value*
Total neutrophils	Group 1	71.9 ± 10.9	0.06
	Group 2	59.9 ± 21.5	
CD66b+ neutrophils	Group 1	78.9 ± 12.9	0.21
	Group 2	83.5 ± 16.4	
CD66b+CD16+CD11a+ neutrophils	Group 1	94.3 ± 12.8	0.15
	Group 2	90.0 ± 19.2	
CD66b+CD16- CD11a+ neutrophils	Group 1	86.9 ± 19.8	0.55
	Group 2	91.4 ± 16.6	
CD66b+CD16- neutrophils	Group 1	6.5 ± 5.1	0.37
	Group 2	14.7 ± 16.7	
CD66b+CD16+ neutrophils	Group 1	93.5 ± 5.1	0.37
	Group 2	85.3 ± 16.7	

SDM: standard deviation from the mean;

* Student’s t test;

** Mann-Whitney U test.

**Table 4:** Comparison of the percentages of monocyte subtypes between patients with head and neck squamous cell carcinoma and healthy volunteers.

Biomarkers	Group	Mean ± SDM	*p* -value
Total monocytes	Group 1	69.6 ± 10.0	0.73*
	Group 2	68.5 ± 6.2	
CD14+ monocytes	Group 1	28.1 ± 34.1	0.13**
	Group 2	8.6 ± 3.4	
CD14+CD86+ monocytes	Group 1	86.2 ± 16.1	0.52**
	Group 2	84.3 ± 13.9	

SDM: standard deviation from the mean;

* Student’s t test;

** Mann-Whitney U test.

Table 5 presents the comparison of the MFI between groups of patients with squamous cell carcinoma and volunteers used to evaluate the expression of subtypes of immune system cells and the activation marker CD69 in these cells. There was no difference between groups for most MFI measurements, except for CD69 expression on CD19+ B lymphocytes and CD56+ NK cells. The MFI of the activation marker CD69 in CD19+ B lymphocytes was higher in cancer patients compared to volunteers (*p* = 0.002). The MFI of CD56+ NK cells was higher in patients with squamous cell carcinoma compared to the group of volunteers (*p* < 0.001).

**Table 5:** Comparison by median fluorescence intensity of immune cell subtypes and expression of the CD69 activation marker between patients with head and neck squamous cell carcinoma and healthy volunteers.

Biomarkers	Group	Mean ± SDM	*p* -value
MFI CD69 in CD3+CD8+ lymphocytes	Group 1	327 ± 253.8	0.50**
	Group 2	188 ± 61.5	
MFI CD69 in CD19+ B lymphocytes	Group 1	532 ± 435.1	0.002**
	Group 2	220 ± 86.2	
MFI CD69 in CD3+CD4+ T lymphocytes	Group 1	343 ± 249.9	0.39**
	Group 2	185 ± 61.8	
MFI CD56+ lymphocytes	Group 1	220 ± 57.8	<0.001**
	Group 2	106 ± 70.3	
MFI CD16 in CD66b+ neutrophils	Group 1	41,112 ± 29,128.2	0.89*
	Group 2	39,913 ± 19,917.7	
MFI HLA-DR in CD66b+CD16+ neutrophils	Group 1	145 ± 46.5	0.64**
	Group 2	143 ± 25.3	
MFI CD11a in CD66b+CD16+ neutrophils	Group 1	1,729 ± 939	0.59**
	Group 2	2,284 ± 782.3	
MFI CD11a in CD66b+CD16- neutrophils	Group 1	2,058 ± 650.8	0.08**
	Group 2	2,048 ± 899.5	
MFI CD86 in CD14+ monocytes	Group 1	4,155 ± 1,503.2	0.30**
	Group 2	3,542 ± 1,081.4	

SDM: standard deviation from the mean;

* Student’s t test;

** Mann-Whitney U test.

## Discussion

With the aim of implementing an immunotherapy protocol for HNSCC patients by inducing immune cell cytotoxicity, Millrud et al.^13^ showed an overall reduction in the number and migratory capacity of leukocytes and a lower rate of their proliferation, which is essential for immunomodulation. A study by Bose et al.^14^ evaluated the status of immunocompetent cells that may participate in the migration to the tumor site. The results showed that all classes of cytotoxic cells are reduced, lowers the death of tumor cells by immune cells and inactivates the cells still available for tumor death, and they also assessed the cytotoxic capacity of neutrophils, monocytes, and lymphocytes in HNSCC patients as compared to healthy individuals. These cells were less cytotoxic for all cancer cells tested as compared to healthy ones. Deregulated immune functions in HNSCC patients directly interfere with nonspecific cytotoxicity mediated by macrophages and NK cells, antibody-dependent cell cytotoxicity, complement-dependent cytotoxicity, and cytotoxic T-lymphocyte reaction^15^-^17^.

Immune protection against tumors can be developed or suppressed from peripheral blood regulatory T lymphocytes^18^, whose activation triggers the expression of early activation surface molecules, such as the CD69 epitope. The initial evaluation stages of T cells are evidenced by the high percentage of TCD69 lymphocytes. HNSCC patients have a higher number of T lymphocytes expressing the CD69 activation marker when compared to healthy individuals^18^,^19^. Functional changes in peripheral blood T lymphocytes in patients with HNSCC can predict disease prognosis^19^,^20^. A high percentage of CD69 T lymphocytes, as measured by flow cytometry, is associated with a poor prognosis. Regulatory T lymphocytes increase their function in the presence of HNSCC, and a higher number of T lymphocytes expressing CD69 is associated with a reduced functional immune system and poor prognosis, with a lower chance of survival^18^,^19^. In our study, it was observed an elevated number of CD4 T lymphocytes and CD19 B lymphocytes expressing the activation marker CD69, suggesting a relationship of this marker with regulating functions of these cells.

CD69 is expressed by monocytes, platelets, CD3 thymocytes, and some peripheral lymphocyte populations, including immune cells from the intestinal mucosa and bronchoalveolar lavage cells, whereas CD69 represents an antigenic activation cell in a wide variety of hematopoietic lineages, including T and B lymphocytes, NK cells, neutrophils, eosinophils, and macrophages. CD69 expression in the thymus seems to be related to the cell maturation process and is restricted to the immature thymocyte T cell receptor. During thymocyte development, CD69 expression is induced at an initial stage of positive selection with a linear relationship between CD69 levels and surface CD3 expression levels. Terminally differentiated thymocytes reduce CD69 expression as soon as they are removed from the thymus, suggesting that CD69 expression by thymocytes is dependent on the thymic environment^18^,^19^.

Resting lymphocytes do not express CD69, which is the earliest activation marker, being rapidly induced on the cell surface after stimulation. A small number of circulating T and NK cells have been also found to be CD69+, most likely as a result of activation^18^,^21^. CD69 expression in T cells requires the RNA and protein synthesis. However, regardless of this synthesis, a description of the presence of CD69 can be identified in the cytoplasm of resting T cells. It seems likely that CD69 expression will enhance T-cell responses after their interactions^18^,^21^.

B lymphocytes can become cancer regulators through an intrinsic mechanism that induces this differentiation. The TGFb produced by regulatory B lymphocytes can convert regulatory CD4 T lymphocytes, which inhibit NK cells and effector CD8 T lymphocytes, blocking the induction of T lymphocyte-mediated tumor immunity and inactivating CD4 T lymphocytes^22^. Cancer-activated B lymphocytes trigger the complete regulatory activity of myelocyte-derived suppressor cells and inhibit TCD4 and TCD8 cells. Tumor regulating B lymphocytes produce TGFb, which acts on monocyte and granulocyte subpopulations by suppressing antitumor CD4+ and CD8+ T cells. B lymphocytes directly lower antitumor immunity through the secretion of IL10 and stimulate the conversion of CD4 T lymphocytes into regulatory T lymphocytes^23^,^24^.

The NK cell can exhibit direct or indirect cytotoxic activity against cells with reduced expression of MHC class I^25^-^27^. It has been observed in some studies that a smaller number of NK cells (CD56) can be found in HNSCC patients. NK cells establish a functional link between innate and adaptive antitumor immunity, sharing some receptors and functions between NK cells and T lymphocytes^28^,^29^. NK cells exhibit characteristics of Th1 and Th2 CD4+ lymphocytes, along with the activities of NK cells and may therefore play a dual role in immune regulation and immunovigilance against tumors. The negative regulation in this NK cell population destroys several cascades of immune signaling in HNSCC patients^30^-^32^. This proportional and functional decrease in immunocompetent cells can be influenced by the positive regulation of regulatory T cells, which are not only involved in the negative regulation of T and NK cells, but also interfere in the presentation of antigens from various cells, including dendritic cells. In our study, it was observed a high percentage of CD56+ NK cells, and the function of these cells must be better explored, as these cells appear having regulators and cytotoxic functions in cancer immune response^33^,^34^.

The elevated number of neutrophils and monocytes observed in HNSCC patients in the study of Bose et al.^14^ does not differ from previous studies in other types of cancers. These data indicate increased inflammation with infiltration of immature neutrophils and monocytes originating from the bone marrow as a result of augmented leukocyte transformation^17^,^35^. An elevated neutrophil / lymphocyte ratio is associated with a greater systemic inflammatory response and a lower survival rate in HNSCC patients. Neutrophils act in a pro-tumorigenic manner by being pro-angiogenic and suppressing adaptive immune responses. At the same time, activated neutrophils have antitumor activity and are associated with increased survival^36^,^37^. A higher number of circulating neutrophils with specific pro-tumor immunophenotyping and a lower number of lymphocytes can be observed in HNSCC patients as compared to healthy individuals^38^. CD14 and CD16 monocytes produce IL10, and, when activated in HNSCC patients, they boost the blood concentration of this interleukin proportionally to the stage of the neoplasm^38^. This increase is associated with a greater chance of survival, indicating that these monocytes have antitumor functions^37^,^38^. There is greater activation of immature macrophages in cancer patients, considering that activated monocytes in patients with advanced neoplasia maintain an immature phenotype^38^. The distribution of monocytes and neutrophils in cancer patients in this study was not altered by the HNSCC, maybe because the advanced stage of the tumor. Monocytes and neutrophils appear to be involved in the earliest stages of immune response in cancer, as observed in previous studies cited below.

The main limitations of this study were the small sample size, the cross-sectional method, selection of patients at more advanced stages of the disease, and inclusion of patients without exposure to previous oncological treatment. The immune response in HNSCC advanced tumors showed an increase in CD4+ and CD56+ cells and a decrease in CD8+ cells. The expression of CD69 activation marker was higher in CD4+ T lymphocytes and CD19+ B lymphocytes. The activity of these cells can be a potential biomarker to evaluate the indication and to monitor response of immunotherapies in cancer. More specific studies are needed including different primary tumor sites, different histological types, staging and carcinogenic agents to evaluate cancer immune response.

## Conclusion

The presence of HNSCC triggers a systemic inflammatory response associated with a decrease in CD8 T cells and an increase in the number of CD4 T and CD56 natural killer cells. CD69 early activation marker was more expressed in CD4+ T cells and CD19+ B cells.

## Data Availability

The data will be available upon request.
